# Characterizing differences in the phosphorus activation coefficient of three typical cropland soils and the influencing factors under long-term fertilization

**DOI:** 10.1371/journal.pone.0176437

**Published:** 2017-05-03

**Authors:** Qihua Wu, Shuxiang Zhang, Ping Zhu, Shaomin Huang, Boren Wang, LinPing Zhao, Minggang Xu

**Affiliations:** 1 Institute of Agricultural Resources and Regional Planning, Chinese Academy of Agricultural Sciences/National Engineering Laboratory for Improving Quality of Arable Land, Beijing, P. R. China; 2 Centre of Agricultural Environment and Resources, Jilin Academy of Agricultural Sciences, Changchun, China; 3 Institute of Plant Nutrition, Resources and Environment, Henan Academy of Agricultural Sciences, Zhengzhou, China; RMIT University, AUSTRALIA

## Abstract

The phosphorus activation coefficient (PAC, the ratio of available P to total P) is an important indicator of soil P availability and the transformation of P fractions. Understanding the details of the PAC is useful to estimate soil available P status and to provide P management guidance. In this research, soils from five long-term (23 years) fertilization treatments in three croplands were selected to examine the relationships between the PAC and P fractions and to analyse the influencing factors. PAC was affected by both soil types and fertilization treatments. Compared to the unfertilized control (CK) treatment, long-term P application significantly increased the PAC, all of the inorganic P (Pi) fractions and most of the organic P (Po) fractions in all the three soils, particularly in chemical fertilizer combined with manure treatment (NPKM). The PAC was significantly correlated to all of the Pi fractions proportions (*P*<0.05) except for Dil. HCl-Pi and Conc. HCl-Pi. Compared with CK, the chemical P and chemical P combined with manure treatments increased the ratio of total Pi fractions to total Po fractions (P_it_/P_ot_); furthermore, NPKM significantly increased the organic C (C_o_) content and decreased the C_o_/P_ot_ ratio. Stepwise multiple regressions showed that PAC = 0.93 C_o_+0.69 P_it_/P_ot_-0.07 C_o_/P_ot_-0.27CaCO_3_-3.79 (R^2^ = 0.924, *P*<0.001). In addition, the variance partitioning analysis showed that more variance of PAC is explained by soil factors (29.53%) than by P input (0.19%) and climate (0.25%) factors. Our findings demonstrate that P application increased the PAC by changing the C_o_ content and the proportion of P fractions. Moreover, soil factors were the most important drivers of P transformations, and NPKM was optimal for improving soil fertility in Chinese croplands.

## Introduction

Phosphorus (P) is an essential nutrient that often limits agricultural plant growth. Both the amount and form of soil P are important considerations for rational P management [1]. Although the total P concentration is high in most soils, only a small fraction of the total P is plant available because P is easily adsorbed on mineral surfaces or bound in solid phases [2]. Therefore, P bioavailability is largely determined by the chemical forms present in the soil [3]. Generally, soil P exists in inorganic P (Pi) and organic P (Po) forms that are present at different amounts and at different ratios depending on the soil type and the management practices used [4]. In some agricultural soils that receive P fertilizer input, large amounts of inorganic P are present [5,6]. However, in soils with low total P content, much of the P is organic because it is cycled within the soil-plant system [7]. Furthermore, the soil Pi and Po are distributed among several geochemical fractions [8,9] with different bioavailabilities that can be transformed under certain conditions [10]. Fractionation schemes using different chemical sequential extractions aid in the examination of different P fractions and soil P dynamics [11,12]. The Hedley sequential fractionation method has been widely used to assess soil P in many conditions: different land-use changes [13], manure application rates [14], and soil profiles [15]. However, few studies have explored the long-term effects of different fertilization treatments on P fractions and availability in variable cropland soils of China.

The total soil P content provides little information regarding P availability for crops, P recovery by crops or P transformation in soils [16]. Available P can be directly taken up by plants and is mainly derived from total P. The ratio of available P to total P is defined as the phosphorus activation coefficient (PAC) and is an important indicator of soil fertility. A high PAC promotes plant growth [17], and the PAC can represent the variations in and the degree of difficulty of the transformations between total P and available P [18]. Many studies have indicated that the transformation between available P and total P is closely related to P fractions and pools. For example, according to coefficient analysis, the relationship between available P and Ca_2_-P was the strongest, followed by Al-P and Ca_8_-P, and the poorest relationship was observed between available P and O-P (P occluded within Fe oxides) [19,20]. Gama-Rodrigues et al. [21] proposed classifications for functional P pools (the most available P pool, the primary mineral pool and the occluded pool) in the soil and identified the processes of P transformations between these pools by using structural equation modelling. However, there is little information regarding the relationships between the PAC and the P fractions and pools. In addition, the PAC was reported can be affected by soil properties [22], climate [13] and P inputs [23], and few researchers have explored the quantitative contributions of these factors and their interactions on the PAC variance.

Black soil (Luvic Phaeozems according to the FAO classification), fluvo-aquic soil (Calcaric Cambisol for FAO) and red earth (Ferralic Cambisols for FAO) are the three soil types used in this study and arise from the northeast, central and south of China, where the main agricultural regions are located. These three soils received five long-term (1990–2013) fertilization treatments (control (CK); chemical nitrogen and potassium (NK); chemical nitrogen, phosphorus and potassium (NPK); NPK plus straw (NPKS); and NPK plus manure (NPKM), the most common fertilizing methods currently used by farmers) at three sites (500 km between each site) [24]. In this study, we addressed the effects of chemical fertilization alone and chemical fertilization combined with manure on (1) PAC characteristics; (2) variations in the concentrations and proportions of P fractions; (3) the relationships between PAC with P fractions and the influencing factors. We expect to first identify important fractions and the mechanisms leading to a high PAC. Second, we will determine the best fertilizer management practices to achieve a high PAC under different cropping systems in China.

## Materials and methods

### Site description

**Fig 1:**
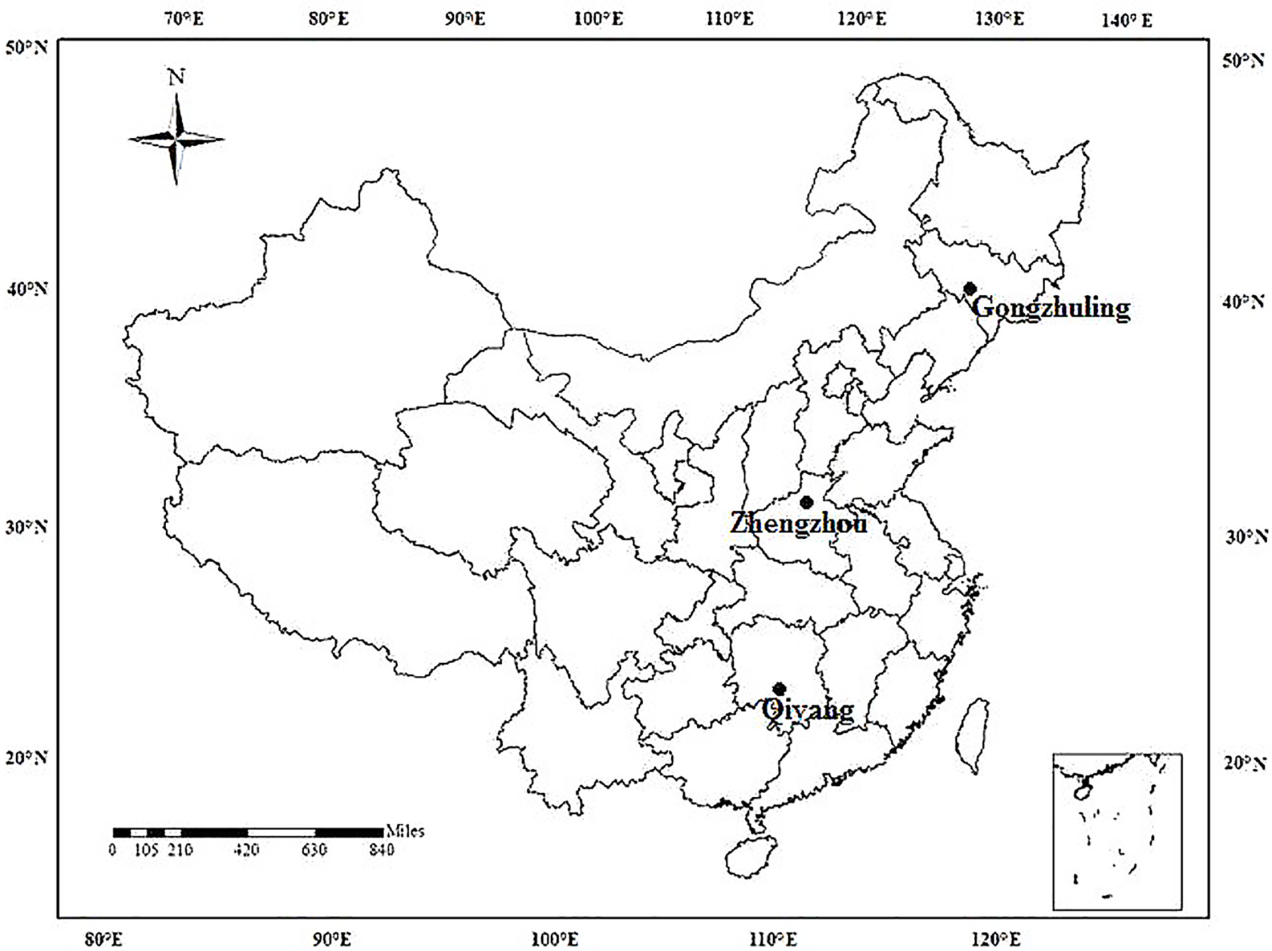
Locations of the three long-term fertilization experimental sites in China.

The three long-term field fertilization sites selected for this study are located in Gongzhuling, Jilin, Northeast China; Zhengzhou, Henan, Central China; and Qiyang, Hunan, Southern China (Fig 1). The soil physicochemical properties in 1990 varied among the sites (Table 1).

**Table 1:** Locations, climate conditions (data are the 1990–2013 means from the China meteorological sharing service system (http://cdc.cma.gov.cn/)) and initial surface soil properties (in 1990) at the three long-term experiment sites.

Parameters	Gongzhuling	Zhengzhou	Qiyang
Altitude (m)	220	21	120
Latitude (N)	43°30′	34°47′	26°45′
Longitude (E)	124°48′	113°40′	111°52′
Mean annual precipitation (mm)	590.7	641	1426.4
Mean annual temperature (°C)	6.6	14.7	18.0
Cumulative effective temperature (>10°C)	2800	5169	5600
Cropping system	Single-cropping, maize	Double-cropping, maize/wheat	Double-cropping, maize/wheat
Soil classification in FAO	Luvic Phaeozems	Calcaric Cambisol	Eutric Cambisol
Soil texture	Clay loam	Light loam	Light loam
Bulk density (g cm^-3^)	1.2	1.5	1.2
Clay (<0.002 mm,%)	32.1	13.4	41.0
Soil pH (soil: water = 1:2.5)	7.6	8.3	5.7
Organic C g kg^-1^)	13.2	6.7	6.6
Total P (g kg^-1^)	0.6	0.6	0.5
Olsen P (mg kg^-1^)	11.8	6.5	4.7

### Cropping practices

Two years before these three long-term fertilization sites were established, the local crops were cultivated as follows without fertilizer to reduce variations in soil fertility between the sites. The cropping systems were different at the three sites and included mono-maize cropping at Gongzhuling (late April to late September) and wheat-maize double-cropping at Zhengzhou (mid-October to early June for wheat and mid-June to late September for maize) and Qiyang (early November to early May for wheat and early April between wheat strips to July for maize). No irrigation was provided to crops at Gongzhuling and Qiyang, but irrigation water was added two or three times to the wheat crop and once to the maize crop at Zhengzhou (approximately 75 mm each time), depending on precipitation amounts. Pesticides were applied during crop growth as needed.

Crops were harvested manually close to the ground, and all of the harvested biomass was removed from the plots with little crop residue return to the land (except in the NPKS treatment). Crop grains were air-dried, threshed, oven-dried at 65°C to a uniform moisture level and then weighed.

### Fertilization treatments

The field experiments were arranged in a randomized block design with 3 replications in ZZ (plot size 45 m^2^), 2 replications in QY (plot size 196 m^2^), and no replications in GZL (plot size 200 m^2^). However, it was possible to divide the individual treatment plots into three sub-plots to capture some spatial variation in our analyses. The following five treatments were assessed in this study: (1) CK (unfertilized control); (2) NK (nitrogen and potassium); (3) NPK (nitrogen, phosphorus and potassium); (4) NPKM (nitrogen, phosphorus, potassium plus farmyard manure), and (5) NPKS (nitrogen, phosphorus, potassium plus maize straw). The annual fertilization rates are summarized in Table 2. At each site, the same amount of total N was applied for the NK, NPK, NPKM and NPKS treatments. The same amounts of inorganic P fertilizer were applied to NPK, NPKM and NPKS treatments, and the same amounts of inorganic K fertilizer were applied to the NK, NPK, NPKM and NPKS treatments. Thus, the NPKM and NPKS treatments received greater amounts of P and K than the NK and NPK treatments because the added manure and straw contained P and K.

**Table 2:** Rates of N, P and K application in the form of chemical fertilizer and manure at the three long-term fertilization sites.

Treatments^a^	Gongzhuling		Zhengzhou		Qiyang	
	inorganic^b^ N-P-K (kg ha^-1^)	organic P (kg ha^-1^)	inorganic N-P-K (kg ha^-1^)	organic P (kg ha^-1^)	inorganic N-P-K (kg ha^-1^)	organic P (kg ha^-1^)
CK	0-0-0	0	0-0-0	0	0-0-0	0
NK	165-0-68	0	353-0-146	0	300-0-100	0
NPK	165-36-68	0	353-78-146	0	300-53-100	0
NPKM^c^	165-36-68	20	353-78-146	19	300-53-100	37
NPKS^d^	165-36-68	3.6	353-78-146	6.9	300-53-100	1.2

a Treatment codes: CK: unfertilized control; NK: nitrogen and potassium; NPK: inorganic nitrogen, phosphorous and potassium; NPKS: NPK plus straw return; NPKM: NPK plus manure.

b Inorganic N fertilizer as urea, P as calcium triple superphosphate, K as potassium sulfate.

c The manures were pig manure from 1990 at Gongzhuling (23.0 Mg ha^-1^ year^-1^) and Qiyang (42.0 Mg ha^-1^ year^-1^) but horse manure from 1990 to 1998 and cattle manure from 1999 to 2013 at Zhengzhou (12.9 Mg ha^-1^ year^-1^). All manure amounts at these three sites were averaged as fresh weight from 1990 to 2013.

d The entire quantity of maize straw was incorporated into the soil at Gongzhuling (approximately 7.5 Mg ha^−1^) and Zhengzhou (on average 6.0 Mg ha^−1^), whereas at Qiyang, half of the maize and wheat straw (approximately 4.5 Mg ha^−1^) was applied.

### Soil sample analyses

Soil samples were collected at a depth of 0–20 cm in September 2013 using a 10 cm diameter soil auger. A total of 9 soil cores from each plot were collected, and 3 cores were combined to form each composite sample. All of the soil samples were air-dried, sieved (2 mm) and stored for analysis.

Soil organic carbon content was determined by vitriol acid–potassium dichromate oxidation [25]. Total P was determined with the H_2_SO_4_-HClO_4_ method [26]. Available phosphorus (Olsen-P) was determined using the Olsen-P method [27]. Soil pH was also measured (mass/volume ratio of 1:2.5; [28]). The CaCO_3_, Fe_2_O_3_ and Al_2_O_3_ were measured following Lu [26].

### P fractionation

To determine the chemical species of P in the soils, the Tiessen and Moir fractionation scheme [9] was used. Briefly, triplicate sub-samples of each soil (1 g) were sequentially extracted as follows: shaking with 30 ml deionized water with a resin strip, which was saturated overnight with bicarbonate ions (NaHCO_3_ 0.5 M, pH 8.5) for 16 h (Resin-P), shaking with 0.5 M NaHCO_3_ at pH 8.5 for 16 h (NaHCO_3_-P), shaking with 0.1 M NaOH for 16 h (NaOH-P), shaking with 1 M HCl for 16 h (Dil. HCl-P), heating with 10 ml concentrated HCl at 80°C in a water bath for 10 min, adding 5 ml 12 M HCl, and then bringing the final volume to 50 ml with deionized water (Conc. HCl-P). Finally, the soil residue was mineralized with concentrated H_2_SO_4_ (300 μl per 30 mg soil residue subsample) at 350°C for 3 h (rate of 4°C/min; Residual-P). Between two consecutive steps, the tubes were centrifuged for 10 min at 25,000×g and 4°C. The supernatant was passed through 0.45 μm cellulose nitrate filters, and the filters were washed with the extractant used in the following step to recover extra soil particles. Both inorganic and organic P (from the difference between the total P content after persulfate digestion and the inorganic P content determined using colorimetry) levels were determined from the 0.5 M NaHCO_3_, 0.1 M NaOH and 12 M HCl extracts.

### Statistical analyses

Analysis of variance was conducted using SAS. Statistically significant differences were determined using the LSD test at *P*<0.05. All of the statistical analyses were conducted using SPSS 20.0. Variance partitioning analysis was conducted with R (R version 3.2.2). All of the data are presented as the average value of three replicates.

## Results

### Soil properties

Changes in the studied properties of the three soil types after different fertilization treatments are shown in Table 3. The soil pH was greatly affected by both the fertilization treatments and soil types. Soil organic C content was higher in treatments with applied P (NPKM, NPKS and NPK) than in treatments without P (NK and CK) for each soil, and the mean organic C concentration was higher at Gongzhuling (15.3 g kg^-1^) than at Qiyang (9.7 g kg^-1^) or Zhengzhou (8.7 g kg^-1^). The Fe_2_O_3_, Al_2_O_3_ and CaCO_3_ concentrations differed slightly among the five fertilization treatments in each site but varied greatly between the three soils. The mean concentrations of Fe_2_O_3_ and Al_2_O_3_ were the highest at Qiyang, and CaCO_3_ was the highest at Zhengzhou (Table 3).

**Table 3:** Selected soil properties for each soil and fertilization treatment. ANOVA significance levels for the effects of soil type, fertilization, and soil type and fertilization interactions.

Treatments^a^	pH	Organic C	CaCO_3_	Fe_2_O_3_	Al_2_O_3_
	H_2_O	g kg^-1^	g kg^-1^	g kg^-1^	g kg^-1^
Gongzhuling					
CK	7.4	13.4	36.5	1.65	1.39
NK	6.0	14.0	22.3	2.54	1.90
NPK	6.0	14.1	22.8	2.23	1.78
NPKM	7.3	21.1	27.3	2.07	1.55
NPKS	7.9	13.7	37.0	1.49	1.46
Zhengzhou					
CK	8.2	6.7	72.8	0.83	0.61
NK	8.1	7.5	76.5	1.12	0.68
NPK	8.0	8.3	77.4	1.23	0.71
NPKM	7.9	10.9	77.2	1.14	0.74
NPKS	8.0	10.2	70.7	1.12	0.69
Qiyang					
CK	5.3	7.5	12.4	3.20	2.14
NK	3.9	6.8	6.1	5.30	2.85
NPK	4.1	9.9	8.8	4.39	2.99
NPKM	6.1	14.3	7.8	2.43	2.28
NPKS	4.2	10.0	15.2	5.86	2.95
*ANOVA*					
Soil type	**	**	**	**	**
Fertilization	**	**	*	*	*
Soil type×fertilization	****	**	**	**	**

a Abbreviations: CK: unfertilized control; NK: nitrogen and potassium; NPK: inorganic nitrogen, phosphorous and potassium; NPKS: NPK plus straw return; NPKM: NPK plus manure.

Significance levels

* represents *P*<0.05 and

** represents *P*<0.01.

### PAC and crop yield

The available P, total P (analyzed by H_2_SO_4_-HClO_4_ method, and independent of sequential fractionations), PAC and crop yield were much higher in treatments with applied P than in treatments without P at each site, especially for NPKM (Table 4). The PAC values in NPKM are 22, 12 and 17 times higher than in CK at Gongzhuling, Zhengzhou and Qiyang, respectively. High crop yields were observed in treatments with high PACs, and Table 4 shows that the highest crop yield was after NPKM or NPKS treatment. The lowest crop yields were in the CK group at Gongzhuling and Zhengzhou. In Qiyang, the maize and wheat yield were 0 for NK because of its very low pH (shown in Table 3).

**Table 4:** PAC and crop yield under different soil types and fertilizations. ANOVA significance levels for the effects of soil type, fertilization, and soil type and fertilization interactions.

Treatments^a^	Available P^b^	Total P^c^	PAC^d^	Maize yield	Wheat Yield
	mg kg^-1^	g kg^-1^	%	t ha^-1^	t ha^-1^
Gongzhuling					
CK	2.9	0.53	0.6	3.44	-
NK	3.4	0.47	0.7	6.61	-
NPK	41.1	0.73	5.6	10.52	-
NPKM	174.2	1.35	12.9	10.71	-
NPKS	34.4	0.8	4.3	10.13	-
Zhengzhou					
CK	2.3	0.6	0.4	4.22	1.31
NK	2.1	0.55	0.4	6.66	1.81
NPK	15.1	0.91	1.7	10.17	5.59
NPKM	53.0	1.12	4.7	10.82	5.76
NPKS	16.2	0.96	1.7	11.57	6.0
Qiyang					
CK	3.9	0.61	0.6	0.06	0.21
NK	4.4	0.49	0.9	0	0
NPK	55.8	1.14	4.9	1.07	0.58
NPKM	181.3	1.76	10.3	4.56	1.45
NPKS	57.3	1.09	5.3	0.78	0.63
ANOVA					
Soil type	**	**	**	**	**
Fertilization	**	**	**	**	**
Soil type×fertilization	**	**	**	**	**

a Abbreviations for the treatments are the same as described in Table 3. There was mono-maize cropping at Gongzhuling and wheat-maize double-cropping at Zhengzhou and Qiyang.

b Available phosphorus extracted by the Olsen method.

c Total phosphorus analysed by H_2_SO_4_-HClO_4_ method, and independent of sequential fractionations.

d Phosphorus activation coefficient.

Significance levels

* represents *P*<0.05 and

** represents *P*<0.01.

### P fractions according to the Hedley procedure

#### Concentrations of P fractions

The concentrations of inorganic P (Pi) fractions were significantly different for each fertilization treatment and soil type (Table 5). Compared to 23 years without P application, long-term P application in the field resulted in a significant increase of Pi fractions (Resin-P, NaHCO_3_-Pi, NaOH-Pi, Dil. HCl-Pi and Conc. HCl-Pi), and the largest increases were from NPKM. Among the three soils, Qiyang exhibited the highest mean concentrations of all Pi fractions except the Dil. HCl-Pi, whereas Zhengzhou had the highest mean concentrations for Dil. HCl-Pi.

**Table 5:** The concentration of different P fractions in each fertilization treatment and soil type (mg kg^-1^). ANOVA significance levels of the effects of soil type, fertilization treatment, and the interactions between soil type and fertilization.

Treatments^a^	Pi^b^ (mg kg^-1^)						Po^b^ (mg kg^-1^)			Total P sum
	Resin	NaHCO_3_	NaOH	Dil. HCl	Conc. HCl	Residual	NaHCO_3_	NaOH	Conc. HCl	
Gongzhuling										
CK	6(1)	3(1)	14(2)	145(27)	120(22)	150(28)	6(1)	79(15)	22(4)	545
NK	10(2)	7(1)	29(6)	61(13)	111(24)	129(28)	15(3)	84(18)	15(3)	462
NPK	53(8)	30(4)	91(13)	109(16)	147(21)	153(22)	8(1)	93(13)	13(2)	697
NPKM	54(4)	135(10)	193(14)	373(28)	212(16)	200(15)	19(1)	136(10)	22(2)	1343
NPKS	34(4)	21(3)	45(6)	274(35)	148(19)	162(21)	7(1)	68(9)	23(3)	781
Zhengzhou										
CK	6(1)	3(1)	5(1)	392(60)	83(13)	120(18)	5(1)	28(4)	9(1)	652
NK	7(1)	7(1)	9(1)	375(59)	85(13)	117(18)	6(1)	25(4)	9(1)	640
NPK	31(3)	47(5)	26(3)	523(58)	105(12)	125(14)	5(1)	27(3)	12(1)	902
NPKM	63(6)	66(6)	34(3)	613(55)	96(9)	156(14)	12(1)	35(3)	32(3)	1108
NPKS	35(4)	50(6)	31(3)	510(56)	90(10)	133(15)	12(1)	39(4)	7(1)	906
Qiyang										
CK	2(0)	4(1)	78(13)	16(3)	301(51)	148(25)	5(1)	33(6)	6(1)	592
NK	12(2)	13(2)	143(26)	12(2)	156(28)	145(27)	13(2)	37(7)	16(3)	548
NPK	75(8)	102(10)	212(21)	72(7)	275(28)	169(17)	14(1)	46(5)	24(2)	987
NPKM	88(5)	269(15)	545(31)	164(9)	332(19)	249(14)	20(1)	65(4)	25(1)	1756
NPKS	81(8)	88(9)	255(25)	73(7)	313(30)	163(16)	12(1)	26(2)	19(2)	1029
ANOVA										
Soil type	**	**	**	**	**	**	**	**	**	**
Fertilization	**	**	**	**	**	**	**	**	**	**
Soil type ×fertilization	**	**	**	**	**	**	**	**	**	**

a Abbreviations for the treatments are the same as described in Table 3.

b Values in parentheses are the proportion (%) of the total soil P (sum of all P fractions), Pi inorganic P, Po organically bound P.

Significance levels

** represents *P*<0.01.

In general, the three Po fractions (NaHCO_3_-Po, NaOH-Po, and Conc. HCl-Po) were the highest after the NPKM or NPKS treatments for each type of soil (Table 5). The Po concentrations were ordered NaOH-Po>Conc. HCl-Po>NaHCO_3_-Po for almost all of the fertilization treatments and soil types. Gongzhuling had the highest mean concentrations of NaOH-Po and Conc. HCl-Po, whereas Qiyang exhibited the highest mean concentration of NaHCO_3_-Po. The fractionated total P (sum of all P fractions) content among the five fertilization treatments for each soil decreased as follows: NPKM>NPKS>NPK>CK>NK.

#### Proportions of P fractions

The different sequential P fractions were classified into three pools [29,30]: (1) labile P (Resin-P+NaHCO_3_-Pi+NaHCO_3_-Po), (2) slowly cycling P (NaOH-Pi+NaOH-Po+Dil. HCl-Pi) and (3) occluded P (Conc. HCl-Pi+Conc. HCl- Po+Residual-P). The proportions of labile P pool were much higher and the proportions of occluded P pool were much lower in applied P treatments, whereas the proportion of slowly cycling P pool only showed small variations among the five fertilization treatments for each soil type (Table 6). The proportions of labile P pool in NPKM were 5, 7 and 11 times higher than in CK at Gongzhuling, Zhengzhou and Qiyang, respectively. The mean proportion of occluded P pool accounted for 53%, 46% and 29% of the total P content in Qiyang, Gongzhuling and Zhengzhou, respectively.

**Table 6:** The percentages of labile, slowly cycling and occluded P pools, the ratio of total Pi to total Po, and the ratio of organic C to total Po in different soil types after different fertilizations.

Treatments^a^	Labile P pool (%)^b^	Slowly cycling P pool (%)^b^	Occluded P pool (%)^b^	P_it_/P_ot_^c^	C_o_/P_ot_^d^
Gongzhuling					
CK	3	44	54	4	125
NK	7	38	55	3	122
NPK	13	42	45	5	124
NPKM	15	52	32	7	119
NPKS	8	49	43	7	140
Zhengzhou					
CK	2	65	32	15	160
NK	3	64	33	15	186
NPK	9	64	27	20	189
NPKM	13	62	26	14	138
NPKS	11	64	25	15	177
Qiyang					
CK	2	21	77	13	169
NK	7	35	58	7	103
NPK	19	33	47	11	118
NPKM	21	44	35	15	130
NPKS	18	34	48	18	178
ANOVA					
Soil type	**	**	**	**	**
Fertilization	**	**	**	**	**
Soil type×fertilization	**	**	**	**	**

a Abbreviations for the treatments are the same as described in Table 3.

b Labile P pool (Resin-P+NaHCO_3_-Pi+NaHCO_3_-Po), slowly cycling P pool (NaOH-Pi+NaOH-Po+Dil. HCl-Pi) and occluded P pool (Conc. HCl-Pi+Conc. HCl- Po+Residual-P) according to De Schrijver et al.[29] and Crews and Brookes [30].

c P_it_ represents the total inorganic P (sum of all Pi fractions and residual P), P_ot_ represents the total organic P (sum of all Po fractions).

d C_o_/P_ot_ is the organic C to the sum of all Po fractions.

Significance levels

** represents *P*<0.01.

The difference between the P_it_ (total inorganic P, sum of all Pi fractions and Residual-P, shown in Table 5) to P_ot_ (total organic P, sum of all Po fractions) (P_it_/P_ot_) ratios was large between fertilization treatments and soils (Table 6). The P_it_/P_ot_ ratios were higher in treatments with applied P than in treatments without P at Gongzhuling and Qiyang, and small variations were observed at Zhengzhou. The organic C (C_o_) to P_ot_ (C_o_/P_ot_) ratio was less than 200 (103–189) in all of the fertilization treatments and soils. Both the mean P_it_/P_ot_ and C_o_/P_ot_ ratios were the highest at Zhengzhou and the lowest at Gongzhuling for the three soils.

### Relationships between the PAC and the proportions of P fractions and soil properties

Correlation analysis revealed that the PAC was positively correlated with the proportions of Pi fractions such as Resin-P, NaHCO_3_-Pi and NaOH-Pi (*P*<0.05) and negatively correlated with the proportions of Residual-P (*P*<0.05), but none significantly correlated with the proportions of Dil. HCl-Pi or Conc. HCl-Pi (Table 7). No significant correlations were observed between the PAC and the proportions of Po fractions. Moreover, the PAC was positively correlated with the proportion of labile P pool (*P*<0.01) and was not significantly correlated with the proportions of slowly cycling P pool and occluded P pool (Table 7).

**Table 7:** Relationships between the PAC with proportions of the P fractions and P pools.

Variable^a^	r	Variable	r	Variable	r
Proportions of Pi fractions		Proportions of Po fractions		Proportions of P pools^b^	
Resin-P	0.58*			Labile P	0.79**
NaHCO_3_-Pi	0.83**	NaHCO_3_-Po	-0.12		
NaOH-Pi	0.51*	NaOH-Po	-0.05	Slowly cycling P	-0.04
Dil. HCl-Pi	-0.25				
Conc. HCl-Pi	-0.15	Conc. HCl-Po	-0.15	Occluded P	-0.31
Residual-P	-0.52*				

a Values are the proportion (%) of the total soil P (sum of all P fractions).

b Classification of P pools is the same as described in Table 6.

Significance levels

* represents *P*<0.05 and

** represents *P*<0.01.

Soil properties have important impacts on P fractions and dynamics, suggesting that PAC is related to soil properties in a complex way. A stepwise multiple regression procedure was used to determine the influences of the independent variables (pH, C_o_, Fe_2_O_3_, Al_2_O_3_, CaCO_3_, P_it_/P_ot_ and C_o_/P_ot_) on the PAC. The linear regression equation was given as follows:

$$\mathrm{PAC}=0.93C_{o}+0.69P_{it}/P_{ot}\;\text{-}\;0.07C_{o}/P_{ot}\;\text{-}\;0.27CaCO_{3}\;\text{-}\;3.79\;\left(R^{2}=0.924,P<0.001\right)$$

The above equation showed that 92.4% of the PAC was controlled by C_o_, P_it_/P_ot_, C_o_/P_ot_ and CaCO_3_. Moreover, the PAC was positively correlated with the C_o_ content and P_it_/P_ot_ value and negatively correlated with the CaCO_3_ content and C_o_/P_ot_ value.

**Fig 2:**
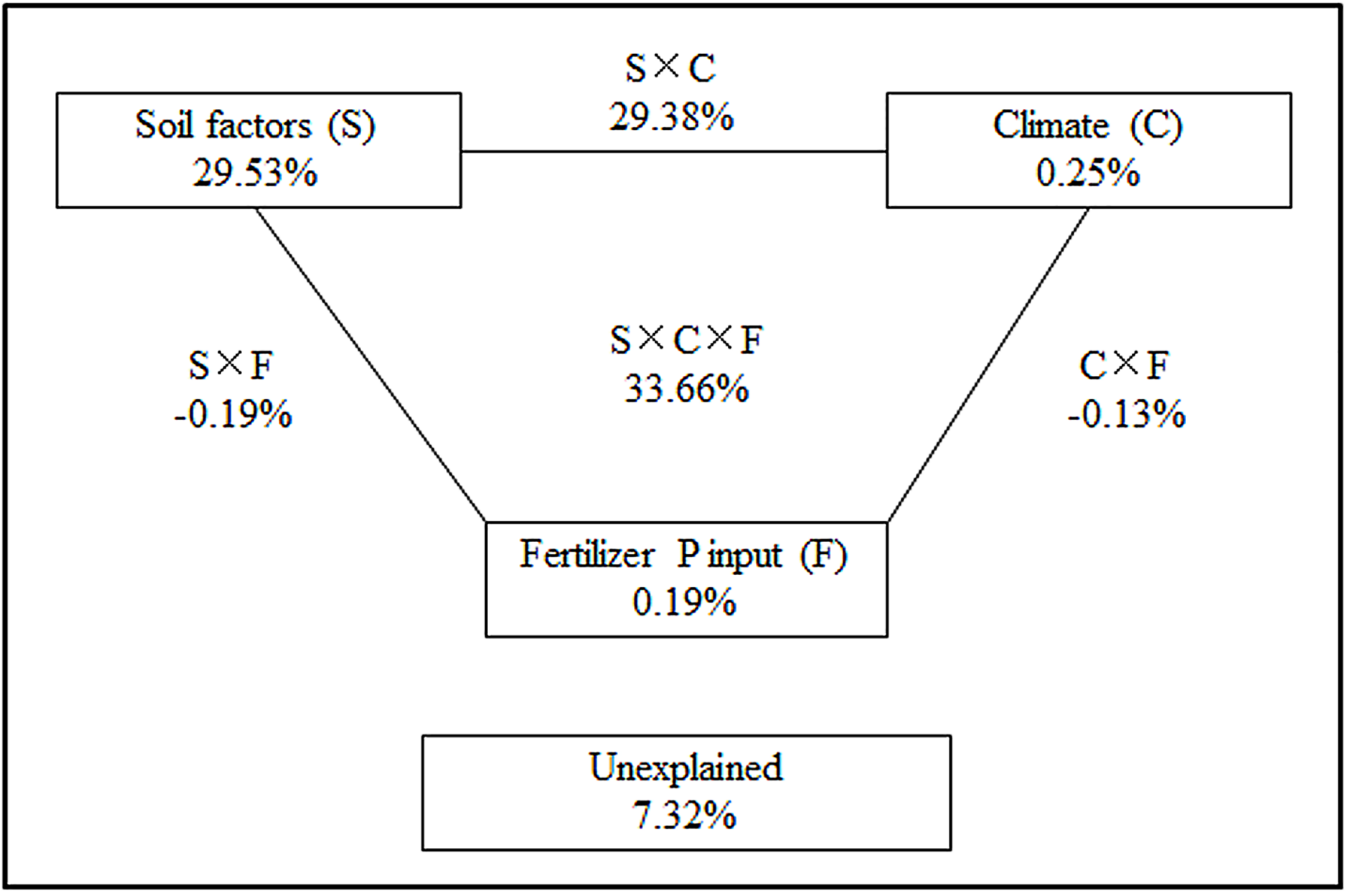
The proportional contributions (%) of the soil factors (S), climate (C), P input (F) and their interactions on the variance of the PAC at the three experimental sites in China based on the variance partitioning analysis method.

To determine the quantitative contributions of soil properties, climate, P inputs and their interactions to the PAC, variance partitioning analysis was used in this study [31]. The soil properties included the organic C, P_it_/P_ot_, C_o_/P_ot_ and CaCO_3_ levels from each fertilization treatment at the three sites (Tables 3 and 6). The total P input included chemical P input and manure and straw P inputs (Table 2). The climate factors included the mean annual temperature, the cumulative effective temperature above 10°C and the mean annual precipitation (Table 1). Among all of the fertilization treatments and sites, 92.68% of total variance of PAC was explained by the three factors (*P*<0.01) and 29.53%, 0.19% and 0.25% of the variance was explained by the soil properties, P input and climate, respectively. The amount of PAC variance explained by the interactive terms of the soil properties, P input and climate was 33.66% (Fig 2, S1 Table).

## Discussion

### Soil P fractions and soil properties

Large variations in each P fraction were observed among the different soils and fertilization treatments in our study (Table 5), which could be explained by different P application rates; soil pH; organic C contents; Fe_2_O_3_, Al_2_O_3_ and CaCO_3_ contents; precipitation or temperatures in the study areas. Many studies have indicated that P fractions in soils are greatly influenced by chemical conditions (pH, organic C), physical properties (particle size, water content), and microorganism and agricultural management practices, particularly the amount of P fertilizer applied [2,32,33]. For instance, Vu et al. [6] examined P fractions in a calcareous soil and found that increasing the long-term (65 years) P application rate significantly increased all of the Pi fractions except for HCl-Pi but did not affect or decrease the concentrations of Po fractions.

Resin-P, NaHCO_3_-Pi and NaHCO_3_-Po are considered very biologically available (labile P) [34]. Compared to CK and NK, the treatments with applied P showed much higher concentrations of the three P fractions, particularly in NPKM (Table 5). Similarly, Crews and Brookes [30] compared the inorganic and organic P fractions in two soils and found that P fractions in the surface layer (0–23 cm) showed almost no change in Broadbalk soils but were depleted in Park Grass soils when unfertilized for more than 100 years; when the soils were fertilized, almost all of the P fractions were enriched. In addition, Song et al. [1] also reported that long-term cultivation without fertilization reduced the soil labile Po content and that the addition of chemical P fertilizer with pig manure increased the labile Po content.

The NaOH-Pi fraction is the inorganic P associated with the exterior of Al and Fe oxides. The NaOH-Po fraction contains stable Po that is associated with the same compounds, and the Dil. HCl-Pi fraction is the stable fraction of Pi bound to Ca [34]. In addition, it has been reported that P fertilizer is rapidly converted from highly soluble P to sparingly soluble amorphous and crystalline P in the soil [6], such as Al-P, Fe-P and Ca-P. The mean NaOH-Pi concentration was highest in Qiyang because of high Fe_2_O_3_ and Al_2_O_3_ soil levels, whereas the Dil. HCl-Pi concentration was highest in Zhengzhou because of the high CaCO_3_ content. The NaOH-Po concentrations were highest in Gongzhuling because of the high organic C content (Table 3). Moreover, the NaOH-Po fraction accounted for the largest percentage of organic P in all soils and fertilization treatments (Table 5), indicating that a relatively high proportion of Po is in stable form and that only a small portion of this pool is biologically active.

### Proportions of P fractions

After long-term fertilization, Pi content became a major component (75–87% in Gongzhuling, 93–95% in Zhengzhou and 88–94% in Qiyang) of P for the different fertilization treatments and soils while the organic P content remained less than 25%. A further comparison showed that the proportion of labile P pool was much higher in treatments with applied P than in treatments without P, and occluded P pool levels followed the opposite trend (Tables 5 and 6). Our results were consistent with other reports, as Dobermann et al. [35] found that the application of P fertilizer mainly increased soluble inorganic P but had little effect on the organic P and residual P fractions. Negassa and Leinweber [11] indicated that long-term cultivation without P fertilizer inputs depleted most of the P fractions, whereas long-term cultivation with P application enriched the P fractions. Overall, agricultural management methods can greatly affect the amounts and forms of soil P [5,30].

The P_it_/P_ot_ (the ratio of total Pi to total Po) ratios differed for each type of soil and fertilization (shown in Table 6). And the P_it_/P_ot_ ratios were higher in treatments with applied P than in treatments without P for the three soils, indicating that the P_it_/P_ot_ ratio reflects fertilization with superphosphate or manure applied to soil. Mcdowell and Stewart [13] also reported that increasing inorganic P fertilizer inputs in the soil increases the inorganic P content relative to the organic P content. In addition, NK displayed a lower P_it_/P_ot_ ratio for the five fertilization treatments because of its higher proportion of Po (Tables 5 and 6), illustrating that considerable soil P was in organic form after long-term cultivation without P application and indicating that inorganic P was more easily absorbed by the crops.

The mean C_o_/P_ot_ ratios were 126, 170 and 140 at Gongzhuling, Zhengzhou and Qiyang, respectively (Table 6). It has been suggested that net P mineralization occurs when C_o_/P_ot_ ratios of <200 [36]. The C_o_/P_ot_ ratios were all <200 in our study, indicating that organic P mineralization could occur. In addition, the C_o_/P_ot_ ratio was lowest for the NPKM treatments at Gongzhuling and Zhengzhou but not at Qiyang (Table 6), which might be related to the obvious differences in manures and soil types. Pagliari and Laboski [37] reported that soil P immobilization was observed after a separated solid manure applied, because of the high ratio of total C to total inorganic P in this manure. In addition, manure organic P includes a fraction that is available to enzyme hydrolysis (Pe) and a fraction that is nonhydrolyzable, and soil clay content can influence the hydrolysis of Pe [38].

### Relationships between the PAC and the proportions of P fractions and soil properties, climate and P inputs

The ratio of available P to total P is defined as the phosphorus activation coefficient (PAC) and can represent the transformations between total P and available P. When the PAC is less than 2.0%, the total P is not easily converted to available P [18]. In the three soils in our study, the PACs were much lower than 2.0% in treatments without P, whereas PACs were greater than 2.0% in most applied P treatments, indicating that P application could increase the PAC and thus total P can easily be converted to available P. Moreover, we found that the PAC was higher for NPKS than NPK at Qiyang and Zhengzhou but was higher for NPK than NPKS at Gongzhuling, which might relate to the climate conditions and soil properties (Table 1).

Soil P is distributed among several geochemical fractions and can be transformed under certain conditions [10]. Different P fractions have different availability [39]. Our results showed that the PAC was correlated with most of the Pi fractions proportions (*P*<0.05) but none of the Po fractions proportions, potentially because Po concentrations were greatly affected by the digestion methods in Hedley sequential fractionation [40]. Furthermore, the PAC was only positively correlated with labile P pool proportions (*P*<0.01) among the three P pools, implying that labile P can easily be transformed to biologically available P [29].

Some studies have demonstrated that the fractions and dynamics of soil P are affected by various soil properties, such as calcium concentrations [41], pH [42], organic matter content and nitrogen concentration [43]. It can be inferred that soil properties also affect the PAC. Less P was absorbed when the organic C content was high [44], leading to a high PAC as shown in Table 4 for NPKM with a high organic C. The PAC was positively correlated with most of the Pi but none of the Po proportions, indicating that PAC was high when the P_it_/P_ot_ ratio was high. In contrast, mineralization was strong when the C_o_/P_ot_ ratio was low, leading to a higher PAC. Higher CaCO_3_ levels in soils might also enhance P retention for the formation and precipitation of Ca-P minerals [45], decreasing PAC.

Soil factors were identified as the most important drivers in the variance of the PAC (Fig 2), indicating that the transformation of total P to available P is greatly affected by soil properties such as the organic C and CaCO_3_ content. Despite the less important roles of climate and P inputs, we found that the amount of variance of the PAC explained by the interactive terms of these three factors was largest (Fig 2), suggesting that climate and P inputs alter the soil factors [46] and subsequently affect the PAC.

## Conclusions

Our results showed that, 23 years long-term P application significantly increased the PAC, all of the inorganic P (Pi) fractions and most of the organic P (Po) fractions in all the three soils, particularly in NPKM. And the PAC differed greatly in different soils and fertilization treatments, indicating that both the two can influence the PAC.

PAC was significantly correlated to most of the Pi fractions proportions (P<0.05) and labile P pool (the sum of Resin-P, NaHCO_3_-Pi, NaHCO_3_-Po) proportions (P<0.01). Moreover, PAC was positively correlated with organic C and the P_it_/P_ot_ value and negatively correlated with the C_o_/P_ot_ ratio. Soil factors were the most important drivers in the variance of the PAC, and the climate and P inputs also showed indirect impacts on the PAC.

High crop yield occurred in treatments with high PAC, such as in the NPKM or NPKS treatments. NPKM had much higher PAC than NPK, and PAC in NPKS was a little higher or lower than in NPK. We conclude that using chemical fertilizers with manure addition is the optimal fertilization method, while climate conditions must be considered for straw return.

## Supporting information

S1 Table Soil properties, climate and P input factors in each fertilization treatment and soil type. (XLS)

## References

[1] Song C, Han X, Wang E. Phosphorus budget and organic phosphorus fractions in response to long–term applications of chemical fertilisers and pig manure in a mollisol. Soil Res. 2011;49: 253–260.

[2] Hinsinger P. Bioavailability of soil inorganic P in the rhizosphere as affected by root-induced chemical changes: a review. Plant Soil. 2001;237: 173–195.

[3] Turner BL, Condron LM, Richardson SJ, Peltzer DA, Allison VJ. Soil organic phosphorus transformations during pedogenesis. Ecosystems. 2007;10: 1166–1181.

[4] Hansen JC, Cade–Menun BJ, Strawn DG. Phosphorus speciation in manure–amended alkaline soils. J Environ Qual. 2004;33: 1521–1527. 15254134 10.2134/jeq2004.1521

[5] McDowell R, Condron L. Phosphorus and the Winchmore trials: review and lessons learnt. N Z J Agric Res. 2012;55: 119–132.

[6] Vu DT, Tang C, Armstrong RD. Changes and availability of P fractions following 65 years of P application to a calcareous soil in a Mediterranean climate. Plant Soil. 2008;304: 21–33.

[7] Sharpley AN. Phosphorus cycling in unfertilized and fertilized agricultural soils. Soil Sci Soc Am J. 1985;49: 905–911.

[8] Hedley MJ, White RE, Nye PH. Plant-induced changes in the rhizosphere of rape (BRASSICA NAPUS VAR. EMERALD) SEEDLINGS. III. CHANGES IN L VALUE, SOIL PHOSPHATE FRACTIONS AND PHOSPHATASE ACTIVITY. New Phytol. 1982;91: 45–56.

[9] Tiessen H, Moir JO. Characterization of available P by sequential extraction In: Carter MR, editor. Soil sampling and methods of analysis. Ann Arbor: Lewis Publishers; 1993 pp. 75–86.

[10] Sharpley AN, Foy B, Withers P. Practical and innovative measures for the control of agricultural phosphorus losses to water: an overview. J Environ Qual. 2000;29: 1–9.

[11] Negassa W, Leinweber P. How does the Hedley sequential phosphorus fractionation reflect impacts of land use and management on soil phosphorus: a review. J Plant Nutr Soil Sci. 2009;172: 305–325.

[12] Condron LM, Newman S. Revisiting the fundamentals of phosphorus fractionation of sediments and soils. J Soils. Sedim. 2011;11: 830–840.

[13] Mcdowell RW, Stewart I. The phosphorus composition of contrasting soils in pastoral, native and forest management in Otago, New Zealand: sequential extraction and ^31^P NMR. Geoderma. 2006;130: 176–189.

[14] Waldrip HM, He Z, Erich MS. Effects of poultry manure amendment on phosphorus uptake by ryegrass, soil phosphorus fractions and phosphatase activity. Biol Fertil Soils. 2011;47: 407–418.

[15] Li M, Zhang J, Wang G, Yang H, Whelan MJ, White SM. Organic phosphorus fractionation in wetland soil profiles by chemical extraction and phosphorus-31 nuclear magnetic resonance spectroscopy. Appl Geochem. 2013;33: 213–221.

[16] Vu DT, Tang C, Armstrong RD. Transformations and availability of phosphorus in three contrasting soil types from native and farming systems: a study using fractionation and isotopic labeling techniques. J Soils Sediments. J Soil Sediment. 2010;10: 18–29.

[17] Chen BL, Sheng JD, Jiang PA. Effect of two types of phosphates on phosphorus efficiency and phosphorus absorption and distribution in cotton field. Journal Xinjiang Agricultural University. 2009;4: 32–37.

[18] Wang JJ, Bai JH, Zhao QQ, Lu QQ, Jia J, Wen X. Profile characteristics of carbon, nitrogen and phosphorus in soils of phragmites australis marshes in Halahai wetlands. Wetland Sci. 2014;12: 690–696.

[19] Wang BR, Xu MG, Wen SL, Li DC. The effects of long term fertilization on chemical fractions and availability of inorgauic phosphate in red soil upland. J Hunan Agric Univ. 2002;28: 293–297.

[20] Shen J, Li R, Zhang F, Fan J, Tang C, Rengel Z. Crop yields, soil fertility and phosphorus fractions in response to long-term fertilization under the rice monoculture system on a calcareous soil. Field Crops Res. 2004;86: 225–238.

[21] Gama–Rodrigues AC, Sales MVS, Silva PSD, Comerford NB, Cropper WP, Gama-Rodrigues EF. An exploratory analysis of phosphorus transformations in tropical soils using structural equation modeling. Biogeochemistry. 2014;118: 453–469.

[22] Shen P, Xu M, Zhang H, Yang X, Huang S, Zhang S, et al Long-term response of soil Olsen P and organic C to the depletion or addition of chemical and organic fertilizers. Catena. 2014;118: 20–27.

[23] Xiao R, Bai J, Gao H, Huang L, Deng W. Spatial distribution of phosphorus in marsh soils of a typical land/inland water ecotone along a hydrological gradient. Catena. 2012;98: 96–103.

[24] He YT, Zhang WJ, Xu MG, Tong XG, Sun FX, Wang JZ, et al Long–term combined chemical and manure fertilizations increase soil organic carbon and total nitrogen in aggregate fractions at three typical cropland soils in China. Sci Total Environ. 2015;532: 635–644. doi: 10.1016/j.scitotenv.2015.06.011 26119378 10.1016/j.scitotenv.2015.06.011

[25] Walkley A, Black IA. An examination of the Degtjareff method for determining soil organic matter, and a proposed modification of the chromic acid titration method. Soil Sci. 1934;37: 29–38.

[26] Lu RK. Analytical methods of soil agricultural chemistry. Beijing, China: China Agricultural Science and Technology Press; 2000.

[27] Olsen SR, Cole CV, Watanabe FS, Dean LA, Olsen SR, Cole CV, et al Estimation of available phosphorus in soils by extraction with sodium bicarbonate. Miscellaneous paper, Institute for Agricultural Research Samaru; 1954.

[28] Thomas GW, Sparks DL, Page AL, Helmke PA, Loeppert RH, Soltanpour PN, et al Soil pH and soil acidity In: Sparks D, editor. Methods of soil analysis. Part II. Madison, WI: Soil Science Society of America; 1996.

[29] De Schrijver A, Vesterdal L, Hansen K, De Frenne P, Augusto L, Achat DL, et al Four decades of post-agricultural forest development have caused major redistributions of soil phosphorus fractions. Oecologia. 2012;169: 221–234. doi: 10.1007/s00442-011-2185-8 22120703 10.1007/s00442-011-2185-8

[30] Crews TE, Brookes PC. Changes in soil phosphorus forms through time in perennial versus annual agroecosystems. Agric Ecosyst Environ. 2014;184: 168–181.

[31] Liang F, Li J, Yang X, Huang S, Cai Z, Gao H, et al Three–decade long fertilization–induced soil organic carbon sequestration depends on edaphic characteristics in six typical croplands. Sci Rep. 2016;6: 30350 doi: 10.1038/srep30350 27492771 10.1038/srep30350 PMC4974611

[32] Blake L, Mercik S, Koerschens M, Moskal S, Poulton PR, Goulding KWT, et al Phosphorus content in soil, uptake by plants and balance in three European long-term field experiments. Nutr Cycl Agroecosyst. 2000;56: 263–275.

[33] Bünemann EK, Steinebrunner F, Smithson PC, Frossard E, Oberson A. Phosphorus dynamics in a highly weathered soil as revealed by isotopic labeling techniques. Soil Sci Soc Am J. 2004;68: 1645–1655.

[34] Tiessen H, Stewart JWB, Cole CV. Pathways of phosphorus transformations in soils of differing pedogenesis. Soil Sci Soc Am J. 1984;48: 853–858.

[35] Dobermann A, George T, Thevs N. Phosphorus fertilizer effects on soil phosphorus pools in acid upland soils. Soil Sci Soc Am J. 2002;66: 652–660.

[36] Wang GP, Liu JS, Wang JD, Yu JB. Soil phosphorus forms and their variations in depressional and riparian freshwater wetlands (Sanjiang Plain, Northeast China). Geoderma. 2006;132: 59–74.

[37] Pagliari PH, Laboski CAM. Dairy manure treatment effects on manure phosphorus fractionation and;changes in soil test phosphorus. Biol Fertil Soils. 2013;49: 987–999.

[38] Pagliari PH, Laboski CAM. Effects of manure inorganic and enzymatically hydrolyzable phosphorus on soil test phosphorus. Soil Sci Soc Am J. 2015;78:1301–1309.

[39] Wang Y, Tang J, Zhang H, Schroder JL, He Y. Phosphorus availability and sorption as affected by long–term fertilization. Agron J. 2014;106: 69–72.

[40] Nascimento CACD, Pagliari PH, Schmitt D, He Z, Waldrip H. Phosphorus concentrations in sequentially fractionated soil samples as affected by digestion methods. Sci Rep. 2015;5: 17967 doi: 10.1038/srep17967 26647644 10.1038/srep17967 PMC4673694

[41] Froelich PN. Kinetic control of dissolved phosphate in natural rivers and estuaries—aprimer in the phosphate buffer mechanism. Limnol Ocean. 1988;33: 649–668.

[42] Turner BL, Cade-Menun BJ, Westerman DT. Organic phosphorus composition and potential bioavailability in semiarid arable soils the Western United States. Soil Sci Soc Am J. 2003;67: 1168–1179.

[43] Canellas LP, Busato JG, Dobbss LB, Baldotto MA, Rumjanek VM, Olivares FL. Soil organic matter and nutrient pools under long–term non–burning management of sugar cane. Eur J Soil Sci. 2010;61: 375–383.

[44] Holfordi ICR, Mattingly GEG. The high- and low- energy phosphate adsorbing surfaces in calcareous soils. Eur J Soil Sci. 1975;26: 407–417.

[45] Shenker M, Seitelbach S, Brand S, Haim A, Litaor MI. Redox reactions and phosphorus release in re-flooded soils of an altered wetland. Eur J Soil Sci. 2005;56: 515–525.

[46] Celi L, Cerli C, Turner BL, Santoni S, Bonifacio E. Biogeochemical cycling of soil phosphorus during natural revegetation of pinus sylvestris on disused sand quarries in northwestern Russia. Plant Soil. 2013;367: 121–134.

